# Standard operating procedure for calculating genome-to-genome distances based on high-scoring segment pairs

**DOI:** 10.4056/sigs.541628

**Published:** 2010-01-28

**Authors:** Alexander F. Auch, Hans-Peter Klenk, Markus Göker

**Affiliations:** 1 Center for Bioinformatics Tübingen, Eberhard-Karls-Universität, Tübingen, Germany; 2 DSMZ – German Collection of Microorganisms and Cell Cultures GmbH, Braunschweig, Germany.

**Keywords:** BLAST, GBDP, GGDC web server, genomics, MUMmer, phylogeny, species delineation, microbial taxonomy

## Abstract

DNA-DNA hybridization (DDH) is a widely applied wet-lab technique to obtain an estimate of the overall similarity between the genomes of two organisms. To base the species concept for prokaryotes ultimately on DDH was chosen by microbiologists as a pragmatic approach for deciding about the recognition of novel species, but also allowed a relatively high degree of standardization compared to other areas of taxonomy. However, DDH is tedious and error-prone and first and foremost cannot be used to incrementally establish a comparative database. Recent studies have shown that *in-silico* methods for the comparison of genome sequences can be used to replace DDH. Considering the ongoing rapid technological progress of sequencing methods, genome-based prokaryote taxonomy is coming into reach. However, calculating distances between genomes is dependent on multiple choices for software and program settings. We here provide an overview over the modifications that can be applied to distance methods based in high-scoring segment pairs (HSPs) or maximally unique matches (MUMs) and that need to be documented. General recommendations on determining HSPs using BLAST or other algorithms are also provided. As a reference implementation, we introduce the GGDC web server (http://ggdc.gbdp.org).

## Introduction

In a recent study [1], we have investigated state-of-the-art methods for inferring whole-genome distances in their ability to emulate DNA-DNA hybridization (DDH), which is the current major technique in microbiology for assessing whether a novel strain can be classified as a species of its own. In almost all groups of *Archaea* and *Bacteria*, a limit of 70% DDH similarity must be under-run to justify the establishment of a new species (see references in [1]). The replacement of DDH by genome-to-genome distances (GGD) is of interest because (i) DDH is cumbersome and is currently carried out in relatively few specialized molecular laboratories only; (ii) distinct DDH methods may differ in their results; (iii) DNA-DNA re-association does not grant access to any information other than the calculated similarity value. In contrast, genome sequence information can of course be re-used in any subsequent comparisons and be explored in multiple ways beyond mere taxonomy.

Algorithms to efficiently determine high-scoring segment pairs (HSPs) or maximally unique matches (MUMs) are valuable tools for inferring intergenomic distances for species delimitation (see [1] and references therein). They correlate well with DDH, are able to cope with heavily reduced genomes and repetitive sequence regions, are very robust against missing fractions of genomic information (depending on the distance formula used), and show a better correlation with 16S rRNA gene sequence distances than do DDH values. The methods work in three main steps, namely the determination of a set of HSPs or MUMs between two genomes, the calculation of distances from these sets, and the conversion of these distances in percent-wise similarities analogous to DDH. The Genome-To-Genome Distance Calculator (GGDC) is a web tool to apply these techniques. It has been devised for, but its use is not restricted to, genome-based species delineation. In the following guideline for conducting and documenting genome distance calculation from sets of HSPs or MUMs, GGDC will serve as a reference implementation.

## Requirements

The GGDC web server (http://ggdc.gbdp.org) uses multi-FASTA files as input. One file per genome is expected, containing each chromosome or plasmid as a single FASTA entry. Alternatively, users can provide a set of Genbank (http://www.ncbi.nlm.nih.gov/) accession numbers. A single query genome can be compared to several reference genomes; organism names can be entered separately. The user can choose between several similarity search tools. Presentation of the results is currently done via an e-mail to a user-specified address. The message also contains a brief explanation of the results.

## Procedure

### Similarity search

**Figure 1:**
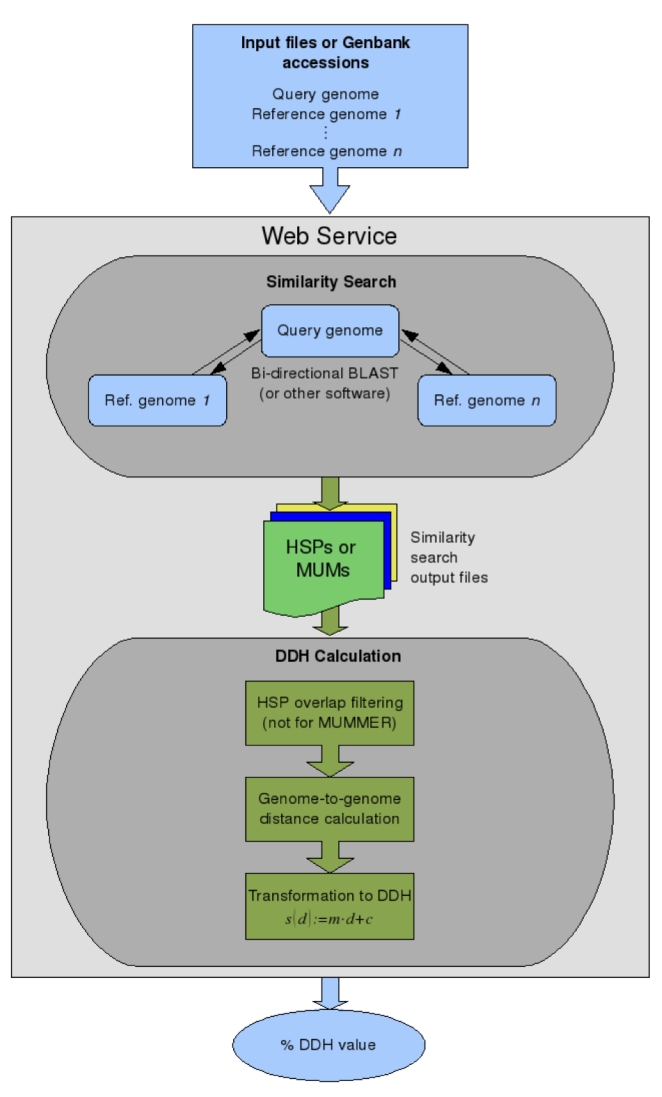
Flowchart outlining the steps required to calculate *in-silico* DDH values. Either Genbank accession numbers or FASTA files are uploaded on the server. The final values are received via e-mail.

Similarities between query and reference genomes are determined by using well-known tools for nucleotide-based sequence similarity search. Currently, NCBI-BLAST [2], WU-BLAST [2], BLAT [3], BLASTZ [4], and MUMmer [5] are available on the web server. Command line parameters for these programs were carefully optimized as documented in [1]. Currently, it is not possible to modify the parameters of these tools via the web interface. An overview of the calculation of in-silico DDH values is provided in Fig. 1.

While we recommend the use of the default settings in general, 'power users' who are interested in and to establishing their own analysis pipelines may want to apply distinct settings. Despite the differences between command line parameters and the algorithms behind those tools, some general propositions can be made as a guideline for advanced users (Table 1, Table 2). Parameters that increase sensitivity also increase run time and memory consumption. Such parameters are the minimum length for a stretch of DNA used as starting point (seed word), the minimal number (or percentage) of identical characters within a match, and the score (or e-value) threshold.

**Table 1:** HSP determination and filtering

**Algorithm**	**WU BLAST^a^**	**NCBI BLAST^b^**	**BLAT^c^**	**MUMmer^d^**	**BLASTZ^e^**
Run time	Very high [M]	Low [M]	High [M]	Very low [M]	Moderate [M]
Memory consumption and output size	High [M]	Moderate [M]	Moderate [M]	Very low [M]	Low [M]
Typical effect on correlation with DDH values	decrease [M]	increase [M]	increase [M]	moderate increase [M]	decrease [M]
**Seed parameter**	W=	-W	-tileSize	-l	T=0 W=
Typical effect on runtime, RAM usage and file size	higher → speedup smaller output files [E]	higher → speedup smaller output files [E]	higher→ speedup; lower → significant increase of memory consumption [M]	higher → speedup smaller output files [M]	higher → speedup smaller output files [E]
Typical effect on correlation with DDH values	N/A	N/A	lower → decrease of correlation [M]	higher → increase of correlation [M]	N/A
**Identity parameter**	score based, i.e., identical to initial word length	score based, i.e., identical to initial word length	-minIdentity	100% (fixed)	score based, i.e., identical to initial word length
Typical effect on runtime, RAM usage and file size	N/A	N/A	insignificant [M]	(none)	N/A
Typical effect on correlation with DDH values	N/A	N/A	lower → increase of correlation [M]	N/A	N/A
**Measure of HSP quality** **used for filtering**	e-value	e-value	substitution score	(makes no sense)	substitution score
Typical effect on subsequent runtime and RAM usage	insignificant [E]	insignificant [E]	lower → small increase of runtime and memory consumption [M]	(none)	lower → small increase of runtime and memory consumption [M]
Typical effect on correlation with DDH values	insignificant [E]	insignificant [E]	lower → small increase of correlation	N/A	higher → slight increase of correlation

The table shows different parameters of the similarity search algorithms and their influence on the correlation with DDH values (for details, see [1]). Note that the best possible correlation of DDH values (similarities) with GGD (dissimilarities) is -1.0; that is, 'high' correlations indicate more negative ones. Seed parameter: Minimum length for a stretch of DNA used as HSP starting point. Identity parameter: Minimum identity within HSP for prolongation. Evidence codes: [M] measured; [E] extrapolated.

^a^Version 2.0MP-WashU [04-May-2006], website http://blast.wustl.edu/. [2]

^b^Version 2.2.18, website ftp://ftp.ncbi.nlm.nih.gov/blast/executables/, [2]

^c^Version 34, website http://users.soe.ucsc.edu/~kent/src/, [3]

^d^Version 3.0, website http://mummer.sourceforge.net/ . [5]

^e^Version 7, website http://www.bx.psu.edu/miller_lab/, [4]

**Table 2:** Command line parameters for similarity search tools as used by the web server. Recommended parameters are in bold.

**Similarity search tool**	**Command line parameter**
NCBI BLAST	blastall -p blastn -i QUERY -d SUBJECT -m 7 -a 1 -S 3 -e 10 **-F 'm D' -b 100000**
WU-BLAST	blastn SUBJECT QUERY mformat=7 cpus=1 E=10 **wordmask=dust B=100000 hspmax=100000** hspsepSmax=50 hspsepQmax=50
BLAT	blat SUBJECT QUERY OUTFILE -t=dna -q=dna -out=blast **-minScore=30 -minIdentity=50**
BLASTZ	blastz QUERY SUBJECT B=2 C=2 **K=3500** Y=700
Mummer	mummer -b -c -F **-l 44 -mum** SUBJECT QUERY

A peculiarity of NCBI-BLAST and WU-BLAST is the usage of filters to mask out regions of low complexity (i.e., repeat filtering) during the seed phase as well as during the extend-phase (when short matches are prolonged) of the algorithm. While it is highly advisable to use filters in the seed phase, resulting in greatly reduced run time, high-scoring pairs may break apart when using filtering during the extend phase. The resulting HSPs have a smaller score (and higher e-value) than a corresponding single HSP would have, and thus, the HSPs may be discarded depending on any score (or e-value) threshold. Thus, when using BLAST to detect orthologous genes, it could be shown that using the filter only in the seed phase ('soft filtering') increases sensitivity [6]. Even when calculating intergenomic distances, a noticeable influence cannot be ruled out since some distance functions use the HSP length as an implicit filtering criterion (trimming procedure, see [7]). The default of NCBI-BLAST is to use the filter for both steps ('hard filtering'), so it is recommended to use the parameters '-F “m D”' ('soft filtering', the filter is only used during the initial phase) when using NCBI-BLASTN, or using '-F ”m S”' with BLASTP, BLASTX and TBLASTX. Corresponding options for WU-blast are 'wordmask=dust' (BLASTN) and 'wordmask=seg' (protein blast). Furthermore, NCBI-BLAST and WU-BLAST limit the number of HSPs that are reported for a given query sequence, by default. This may be acceptable for small queries, but it is not when using whole genomic sequences. In contrast to NCBI-BLAST, WU-BLAST allows to entirely dispose of any limitation for the amount of HSPs, but it has to be considered that this leads to a severe increase in memory consumption. Hence, we propose to set a limit of 100,000 HSPs, which should be sufficient to cover all matches even for highly similar genomes, while memory usage remains feasible (NCBI-BLAST: '-b 100000', WU-BLAST: 'B=100000 hspmax=100000'). When using WU-BLAST, the parameters 'hspsepSmax' and 'hspsepQmax' should be set to avoid the linkage of distant HSPs. This improves running time without affecting sensitivity. A threshold of 50 is sufficient for genomic sequences.

**Figure 2:**
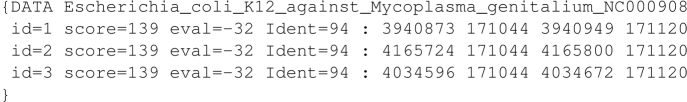
Example of a CGVIZ file. The e-value is stored using its logarithmic value (base 10).

HSPs (or MUMs) are determined by performing similarity searches for each combination of query genome and reference genome. Due to the asymmetric nature of heuristic similarity search strategies, the search is performed twice, first using the reference genome as 'subject sequence' and the query genome as 'query sequence', and second, using the reference genome as 'query sequence' and the query genome as 'subject sequence'. The HSPs (or MUMs) are stored in condensed form using the CGVIZ format [8], which comprises the start and stop coordinates of the matches together with statistical data (e-value, score, alignment length, and percentage identical characters for HSPs, alignment length for MUMs, see Figure 2). The resulting data is sufficient for the distance calculation, while preserving storage space.

### Distance calculation

Distances between genomes are calculated using GBDP as described in [7,9,10]. When using NCBI-BLAST, WU-BLAST, BLAT, and BLASTZ, the greedy-with-trimming algorithm [7] is applied using distance functions (1), (2), and (3) (see [1]). Distances for MUMmer are calculated using the coverage algorithm [7] with distance function (1). These settings currently can not be modified via the web interface. Considering error ratios and correlation with DDH (see [1]), we recommend distance functions (2) or (3) for all similarity search algorithms except MUMmer, for which distance function (1) should be used. For the 'power user', an overview of our propositions regarding HSP/MUM overlap filtering and distance calculation is provided in Table 3.

**Table 3:** HSP overlap filtering and distance calculation.

**HSP overlap filtering**			
Algorithm/implementation	no filtering (coverage functions, see [1])	HSP overlap filtering using the greedy-with-trimming approach described in [1]	
Typical effect on correlation with DDH values	decrease (except for MUMmer) [M]	increase [M]	
			
**Distance calculation**			
**Ratio**	**Length HSPs per total ** **genome length (1)**	**Number of identities within** **HSPs per total HSP length (2)**	**Number of identities** **within HSPs per total** **genome length (3)**
Typical effect on correlation with DDH values	increase [M]	moderate decrease; considerable decrease for MUMmer [M]	increase [M]
**Estimation of total ** **genome length**	**Minimum**	**Average**	
Typical effect on correlation with DDH values	insignificant [M]	insignificant [M]	
**Use logarithm**	**Yes**	**No**	
Typical effect on correlation with DDH values	no effect [M]	no effect [M]	

Evidence codes: [M] measured; [E] extrapolated.

Filtering of HSPs having an e-value above 10^-2^ should be applied for BLAT, NCBI-BLAST and MUMmer prior to distance calculation, while it is not necessary for BLASTZ and WU-BLAST. A downstream filtering step has the advantage that it can easily be changed without the necessity to re-run the costly similarity search with adapted parameters. This enables one to reuse the data for further processing.

### Conversion to percent-wise similarities

The obtained distance values *d* are converted into percent-wise similarities *s*(*d*) by using the corresponding values for intercept *c* and slope *m* according to Table 4:


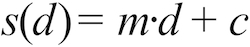


**Table 4:** Distance thresholds and conversion values.

**Method**	**Distance** ** function**	**Distance** **threshold^1^**	**Error-ratio**	**Intercept (*c*)**	**Slope (*m*)**
NCBI BLAST	Trimming (1)	0.2676	0.0860	96.8979	-121.4848
	Trimming (2)	0.0412	0.0430	90.3998	-438.3134
	Trimming (3)	0.2945	0.0860	98.6313	-118.8770
WU-BLAST	Trimming (1)	0.0436	0.2796	122.9402	-406.2128
	Trimming (2)	0.0870	0.0430	82.1068	-166.2293
	Trimming (3)	0.2870	0.0860	115.4105	-191.9086
BLAT	Trimming (1)	0.2672	0.0753	97.7166	-127.9852
	Trimming (2)	0.0416	0.0430	87.0748	-376.3038
	Trimming (3)	0.2811	0.0645	100.6280	-122.5151
BLASTZ	Trimming (1)	0.2389	0.2043	89.8757	-102.0887
	Trimming (2)	0.0575	0.0538	85.1650	-273.1803
	Trimming (3)	0.3344	0.1828	111.9235	-125.1989
MUMmer	Coverage (1)	0.6110	0.0430	130.9618	-116.4258

1 - Analogous to 70% DDH

The percent-wise similarity *s*(*d*) can be used analogous to a DDH value. Values for intercept and slope are determined by applying the robust line fitting procedure as implemented in the R package (Version 2.6.2 [11], ) to the dataset described in [1] (or any subsequently enlarged collection of DDH values and corresponding genomes).

Additionally, the corresponding distance threshold as determined in [1] can be used for species delimitation. Any distance value above the threshold can be regarded as indication that the two genomes analyzed represent two distinct species.

### Recommended use of the server and interpretation of the results

The default similarity search program on the web server is currently NCBI-BLAST, which appears both reasonably fast and reasonably accurate (Table 1). Use of BLAT resulted in somewhat higher correlations with DDH values from literature [1] but takes more time to complete. We thus recommend NCBI-BLAST for testing and for large datasets and BLAT for the final analysis of a small number of genomes.

The e-mail sent to the user includes the results for all three distance formulas. Considering error ratios at 70% DDH, we recommend formula (2). This formula must be used if incomplete genome sequences are submitted to the server [1]. If the overall correlation with DDH is of interest, and particularly if the 70% threshold is less relevant, we strongly recommend formula (3) and the correlations-based DDH estimates.

## Implementation

The GGDC web site is built using PHP 5.0. Submitted jobs are delegated to a pipeline script implemented using BASH and TCL. The pipeline script loads necessary files from Genbank, either invokes the chosen similarity search tool directly (when choosing MUMmer), or uses the GBDP application, which has built-in support for NCBI-BLAST, WU-BLAST, BLASTZ, and BLAT. After calculating distances using the GBDP application, the script processes the output, calculates the corresponding similarity percentage, and sends the data to the user as e-mail. A cleanup script deletes old job files after 24 hours.

The GBDP application is implemented in Java. The current version calculates all distances concurrently, which are output in Phylip and Nexus format suitable for post-processing by phylogeny applications. The GBDP application is available as a stand-alone tool by request from auch@gbdp.org.

New versions of the GBDP application and similarity search tools will be incorporated in the future, when available.

## Remarks

Methods for whole-genome distances other than HSP-based or MUM-based ones can be devised (see overview in [12]), but have not yet been tested in the context of bacterial and archaeal species delineation. It is our intention to keep improving the distance calculation and the DDH estimation by augmenting the existing software and the empirical dataset used for benchmarking and by adding and assessing novel methods.

## References

[1] Auch AF von Jan M Klenk HP Göker M Digital DNA-DNA hybridization for microbial species delineation by means of genome-to-genome sequence comparison. Stand Genomic Sci 2010; 2: 117-134 10.4056/sigs.531120 PMC3035253 21304684

[2] Altschul SF Gish W Miller W Myers EW Lipman DJ Basic local alignment search tool. J Mol Biol 1990; 215: 403-410 2231712 10.1016/S0022-2836(05)80360-2

[3] Kent WJ BLAT – the BLAST-like alignment tool. Genome Res 2002; 12: 656-664 11932250 10.1101/gr.229202 PMC187518

[4] Schwartz S Kent WJ Smit A Zhang Z Baertsch R Hardison RC Haussler D Miller W Human-mouse alignments with BLASTZ. Genome Res 2003; 13: 103-107 10.1101/gr.809403 12529312 PMC430961

[5] Kurtz S Phillippy A Delcher AL Smoot M Shumway M Antonescu C Salzberg SL Versatile and open software for comparing large genomes. Genome Biol 2004; 5: R12 10.1186/gb-2004-5-2-r12 14759262 PMC395750

[6] Moreno-Hagelsieb G Latimer K Choosing BLAST options for better detection of orthologs as reciprocal best hits. Bioinformatics 2008; 24: 319-324 10.1093/bioinformatics/btm585 18042555

[7] Henz SR Huson DH Auch AF Nieselt-Struwe K Schuster SC Whole-genome prokaryotic phylogeny. Bioinformatics 2005; 21: 2329-2335 10.1093/bioinformatics/bth324 15166018

[8] Delgado-Friedrichs O Dezulian T Huson DH A meta-viewer for biomolecular data. GI Jahrestagung 2003; 1: 375-380

[9] Auch AF Henz SR Holland BR Göker M Genome BLAST distance phylogenies inferred from whole plastid and whole mitochondrion genome sequences. BMC Bioinformatics 2006; 7: 350 10.1186/1471-2105-7-350 16854218 PMC1564419

[10] Auch AF, Henz SR, Göker M. Phylogenies from whole genomes: Methodological update within a distance-based framework. German Conference on Bioinformatics, 2006. Available online, urn:nbn:de:bsz:21-opus-34178

[11] The R project for Statistical Computing http://www.r-project.org

[12] Klenk HP Göker M En route to a genome-based classification of *Archaea* and *Bacteria*? Syst Appl Microbiol (Submitted). 20409658 10.1016/j.syapm.2010.03.003

